# Will animal reservoirs give us the next SARS-CoV-2 variant?

**DOI:** 10.1371/journal.ppat.1014008

**Published:** 2026-03-03

**Authors:** Davey Smith

**Affiliations:** University of California San Diego School of Medicine, San Diego, California, United States of America; University of Iowa, UNITED STATES OF AMERICA

## 1. What does it mean that SARS-CoV-2 is now a virus with multiple natural hosts?

Like all known human coronaviruses, SARS-CoV-2 originated from animals, most likely through wildlife sold at the Huanan Seafood Market, with bats serving as the deeper evolutionary reservoir [1]. Since its emergence in humans, SARS-CoV-2 has repeatedly crossed into non-human hosts. Over five years of widespread circulation, the virus has been detected in a surprising array of animals, including white-tailed deer, mink, rats, hamsters, horses, cats, zoo animals and dogs, though sustained transmission is clearly documented only in deer and farmed mink, with other species showing limited or no onward spread. For white-tailed deer and mink, the virus has achieved sustained animal-to-animal transmission and spillback to humans [2–6].

This shift transforms SARS-CoV-2 from a purely human epidemic into a network of linked epidemics across species. Humans remain their largest host, but no longer its only long-term home. When a virus gains multiple such homes, it also gains more ecological space and evolutionary possibilities. This article does not address the original zoonotic emergence of coronaviruses broadly; instead, it focuses on the less explored problem: *how sustained circulation of SARS-CoV-2 in animal reservoirs may shape future human disease.*

## 2. How does a spillover host differ from a true reservoir?

Spillover hosts get infected but do not maintain the virus without frequent human introductions. Cats, hamsters, many zoo animals, and most livestock fall in this category. They illustrate SARS-CoV-2’s broad host range, but they do not sustain independent epidemics.

A reservoir, in contrast, supports stable transmission within its own population and can periodically send the virus back into humans or into other animals. White-tailed deer now meet this definition: they show widespread natural infection, high seroprevalence, and months of deer-to-deer spread across large regions [5,7]. Mink in farm settings functioned as high-amplification, temporary reservoirs before large-scale depopulation curtailed sustained circulation. These outbreaks allowed viral evolution within mink populations with return-adapted lineages to humans [8].

This distinction matters because reservoirs give the virus time. Time allows genetic drift, host adaptation, and long evolutionary excursions that may shape the virus away from the constraints of human immunity.

## 3. How did SARS-CoV-2 spread from humans into animals?

Most documented non-human SARS-CoV-2 infections began with infected people. Infected workers introduced the virus into mink barns; hikers, hunters, and suburban residents seeded it into deer; pet owners infected their animals; keepers infected zoo carnivores and primates [3]. Once SARS-CoV-2 entered a species with dense social contact or seasonally tight aggregations, animal-to-animal transmission could take off. People are not the only social mammals.

Some species provided fertile ground. Deer host high ACE2 and TMPRSS2 expression in the upper airway and often congregate in groups where respiratory viruses travel easily [8,9]. Mink farms created crowded, temperature-controlled environments ideal for viral spread. These factors turn occasional human-to-animal spillover into sustained animal epidemics. Representative spike mutations associated with deer- and mink-derived SARS-CoV-2 lineages, mapped to spike protein domains and contrasted with mutations common in human transmission (Table 1).

**Table 1:** Key spike mutations in deer- and mink-derived SARS-CoV-2.

Mutation	Primary animal host(s)	Spike region	Supported functional effect	Established in human lineages?	Key references
**Y453F**	Mink	RBM	Increases binding to mink ACE2; modest reduction in neutralization by some human mAbs and convalescent sera; improves replication in mink	No sustained human circulation; limited mink-to-human spillover only	Refs [10–12]
**F486L**	Mink, WTD	RBM	Convergent animal adaptation; improves infection of mink; minimal effect on human serum neutralization	Yes, but rare, sporadic human cases only; not in VOCs	Refs [8,11,13]
**N501T**	Mink, WTD	RBM	Enhances ACE2 binding in mustelids and cervids; reduces fitness in human airway cells compared with N501Y	Extremely rare in humans; not established	Refs [11,14]
**Δ69–70**	Mink (Cluster 5)	NTD	Increases infectivity; alters NTD antigenicity; same deletion later selected independently in humans	Yes (Alpha, Omicron lineages)	Refs [12,15]
**Δ143–145**	WTD	NTD antigenic loop	Deletes NTD neutralizing epitope; analogous to immune-escape deletions in Alpha and Omicron	Yes (near-identical deletions in VOCs)	Ref [14]
**L452M**	Mink	RBM	Co-occurs with F486L; likely compensatory for ACE2 binding and spillback fitness	No (humans evolved L452R/Q instead)	Ref [8]
**Q314K**	Mink	S1 (SD2)	Compensatory mutation paired with F486L; inferred structural stabilization	No	Ref [8]
**I692V**	Mink (Cluster 5)	S2 (post-cleavage region)	Likely host-adaptive; may reduce human cell entry when combined with other mink mutations	No	Ref [12]
**M1229I**	Mink (Cluster 5)	Transmembrane domain	Part of mink-specific spike constellation; no demonstrated antigenic effect alone	No	Ref [12]
**E484D**	WTD in B1.596 lineage	RBM	Mediates ACE2-independent entry	Yes, but rare (<0.5% globally)	Refs [16–18]
**L18F**	WTD	NTD	Associated with escape from NTD-binding antibodies; recurs in independent deer clusters	Yes. Present in Delta; associated with reduced antibody neutralization	Ref [19]
**H245Y**	WTD	NTD	Unknown	Yes. Observed in B.1.2 deer lineages; rare in humans	Ref [16]
**L1035F**	WTD	NSP3	Strong candidate for deer-specific adaptation	Yes. Emerged in humans prior to deer outbreaks, but rare in humans (<0.05%)	Refs [19,20]
nsp1 (82-86), nsp2_T434I, nsp2_P597L, nsp12_A382V, nsp13_M474I	WTD	Non-Spike: ORF1ab	Strong candidate for deer-specific adaptation	Yes, but rare in humans (<0.05%)	Ref [16]

ACE2, angiotensin-converting enzyme 2, the cellular receptor for SARS-CoV-2; RBD, receptor-binding domain of spike; RBM, receptor-binding motif within the RBD that directly contacts ACE2; NTD, N-terminal domain of spike; S1, spike subunit 1 containing receptor-binding regions; SD2, subdomain 2 of S1; S2, spike subunit 2 responsible for membrane fusion; HR2, heptad repeat 2 region within S2 involved in fusion; TM, transmembrane domain anchoring spike in the viral envelope; Δ, deletion of one or more amino acid residues; mAb, monoclonal antibody; NSP, Nonstructural Protein; WTD, White Tail Deer; VOC, Variant of Concern as defined by WHO; spillback, transmission of SARS-CoV-2 from an animal reservoir back into humans.

## 4. Which animals matter most for future human spillback?

The clearest reservoirs today are white-tailed deer and farmed mink [21], with deer sustaining long chains of transmission that often preserve older human variants [14]; in some regions, deer still carry Alpha-like lineages long after those variants disappeared from nearby humans, and these viruses evolve faster in deer, accumulating distinctive mutational patterns that let deer serve as both amplifiers of active strains and “cold archives” of extinct ones. Mink remain important where farming persists, as outbreaks sweep rapidly through barns, generate mink-adapted spike mutations, and have produced multiple spillback events in workers [8]. Rats occupy a watchful middle ground: they are experimentally susceptible to several variants, some urban populations show signs of natural infection. Wastewater sequencing has revealed highly divergent viral genomes whose sources remain unresolved, but these data do not demonstrate infectious transmission or confirm animal reservoirs [22]. Other species such as cats, hamsters, and zoo carnivores can transmit SARS-CoV-2, but none have shown sustained, independent viral maintenance in the wild [23,24]. Of course, time may tell.

## 5. How does SARS-CoV-2 evolve once it settles into animal reservoirs?

Once SARS-CoV-2 enters a new animal host, the host’s biology shapes the virus. Different animals express ACE2 and related entry factors in different tissues. Their innate immune responses vary. Their MHC molecules present different viral peptides. Together, these factors place the virus under selection pressures that do not exist in humans.

Several patterns now stand out:

- Deer lineages evolve rapidly. Deep sequencing shows accelerated divergence and mutations that rarely appear in human viruses [25]. High seroprevalence and evidence of reinfection in white-tailed deer suggest that SARS-CoV-2 is now circulating in populations with substantial pre-existing immunity, shaping viral evolution in ways distinct from naïve hosts.
- Mink outbreaks produce characteristic spike mutations. Some of these reduce neutralization by human sera, though modestly [8].
- Cryptic wastewater lineages show rodent-adaptive signatures. They carry constellations of receptor-binding mutations that improve infection of mouse or rat cells in vitro [26]. It remains unclear if these data show infectious transmission, however.

Animal reservoirs allow the virus to explore genetic combinations that human infections seldom permit. Importantly, animals do not select for preservation of epitopes that matter to human immunity. Mutations that erode such epitopes may drift or hitchhike along with host-adaptive changes. Over many transmission cycles, this can create lineages that sit antigenically far from those currently circulating in humans [5].

## 6. Do we already have evidence of animal-derived viruses infecting humans?

Yes. The best example comes from Canada. In Ontario, researchers identified a highly divergent lineage in free-ranging deer that differed from the original Wuhan strain at dozens of positions and carried substitutions associated with non-human hosts. A closely related virus appeared in a human with documented deer exposure, consistent with deer-to-human spillback [14]. Mink-to-human transmission has been documented repeatedly in farm outbreaks [2,8,21]. A pet-shop outbreak in Hong Kong traced back to infected hamsters shows that rodent-derived transmission can occur under real-world conditions [23].

So far, no animal-derived lineage has sparked a new global human wave of *SARS-CoV-2*. But these events demonstrate that spillback is not theoretical.

## 7. Could an animal-adapted lineage escape today’s human immunity?

This would not occur through a single mutation or over a short timescale. The concern lies in long-term divergence. Human immunity today is shaped by repeated exposure through vaccination or infection to Omicron-like spikes. Animals do not apply that same selective landscape. In reservoirs like deer, changes that undermine Omicron-focused human immunity may accumulate simply because they do not affect transmission in deer [5]. Evolution of SARS CoV 2 variants (e.g., alpha, delta) that carry greater pathogenicity for humans raises the possibility that a future spillback could prove deadlier than the Omicron variants now in circulation [27].

If such a lineage spills back into humans after extensive divergence, existing antibodies may bind poorly, neutralization may drop, and vaccines tuned to recent human strains may protect less well. This would not create a “brand-new” virus, but it could behave like a significantly shifted version of SARS-CoV-2 from the perspective of human immunity.

This pattern mirrors the ecology of influenza A viruses, which circulate in birds and swine, periodically re-entering humans as antigenically shifted strains. The 2009 H1N1 emergence and more recent H3N2v outbreaks illustrate how long-term evolution in animal reservoirs can reintroduce variants with altered pathogenicity into immunologically primed human populations [28–31].

## 8. How can we detect dangerous spillback early enough to act?

Early warning requires looking in the right places. Three practical pillars stand out:

- **Track animals in likely reservoirs.** Routine testing and sequencing in deer, mink farms where they still operate, and targeted rodent surveillance in high-risk urban areas [32].
- **Track unusual lineages in wastewater.** Wastewater sequencing can reveal divergent viral lineages long before clinical sampling does. Following up on those signals helps locate the animal or human sources [26].
- **Share data across human, veterinary, and environmental sectors.** A One Health approach is the only workable system for a virus that crosses species so freely [33,34].

Together, these steps create a surveillance net that can detect reservoir-derived lineages before widespread human transmission begins.

## 9. Why should pathogen researchers care, even if they do not work on coronaviruses?

Animal reservoirs slow down viral extinction. They expand the virus’s evolutionary playground. They give SARS-CoV-2 places to mutate where human immunity has little influence. For virologists and pathogenesis researchers of any specialty, these reservoirs show how quickly an emerging virus can become an ecological resident [3]. Ignoring the animal side of SARS-CoV-2 means accepting surprise when spillback occurs. Paying attention gives us a chance to see the next jump coming, and maybe even prevent it.

### Use of AI

Used ChatGPT to edit and check the grammar of the manuscript.
